# The Correlation Analysis Between Salary Gap and Enterprise Innovation Efficiency Based on the Entrepreneur Psychology

**DOI:** 10.3389/fpsyg.2020.01749

**Published:** 2020-08-04

**Authors:** Xingchen Pan, Xiangyu Wan, Haoran Wang, Yingji Li

**Affiliations:** ^1^Graduate School, Gachon University, Seongnam, South Korea; ^2^Institute of Quantitative and Technical Economics, Chinese Academy of Social Sciences, Beijing, China; ^3^Graduate School of Pan-Pacific International Studies, Kyung Hee University, Seoul, South Korea; ^4^School of Management, Chongqing Institute of Engineering, Chongqing, China

**Keywords:** salary gap, enterprise innovation efficiency, entrepreneur psychology, senior managers, ordinary employees

## Abstract

Under the analysis of the psychological level of entrepreneurs, the study focuses on discussing the relationship between the salary gap and enterprise innovation efficiency. It provides a reference basis for promoting employee innovation activities and improving the market competitiveness of enterprises. First, the role of entrepreneur psychology in decision-making was explained theoretically. Based on this, the relationship between the salary gap of employees and enterprise innovation was analyzed from a theoretical perspective. In the empirical analysis, this paper selected the data of China’s A-share manufacturing listed enterprises from 2012 to 2016 as the research sample. The explanatory variables include the salary of senior management, the salary of the ordinary employee and salary gap. The explained variable is enterprise innovation efficiency. By constructing an econometric model, this study used a multiple regression model to empirically analyze the correlation between the salary gap and innovation efficiency. The regression coefficients between monetary salary and equity salary of senior managers and enterprise innovation performance are 5.545 and 1.003, respectively. The regression coefficient between the salary of the ordinary employee and enterprise innovation efficiency is 8.357. The regression coefficient between the internal salary gap of the senior management team and the enterprise innovation efficiency is 3.552, both of which show a significant positive correlation at the 1% level. The regression coefficient between the salary gap between senior managers and ordinary employees and the enterprise innovation performance is −3.032, which is significantly negatively correlated at the 5% level. The internal salary gap of the senior management team has a significant positive effect on enterprise innovation efficiency. The salary gap between senior managers and ordinary employees has a negative effect on enterprise innovation efficiency. Enterprises should optimize the salary structure from two levels of senior managers and ordinary employees, to stimulate the work enthusiasm of employees at all levels, and promote enterprise innovation.

## Introduction

Since the reform and opening-up, China’s economy has developed rapidly and China has become the world’s largest manufacturing country. At the same time, however, China’s manufacturing is still dominated by low-end products in batches, and there is still a large gap compared with high-tech manufacturing in developed countries. As the foundation and lifeblood of China’s national economy, the manufacturing industry is the key supporting force to promote scientific and technological progress and the main direction of technological innovation (Hou, 2018). Accordingly, in order to keep the enterprise stable in the market for a long time, it is necessary to reflect its market competitiveness through innovative methods. In recent years, China has attached great importance to the improvement of manufacturing innovation capabilities at the national level. Both the outline of science and technology development as well as the reform of the science and technology system have clearly pointed out that innovation is the primary driving force for development. The country should continue to work toward the goal of “becoming a strong country in science and technology innovation by 2050” (Gu and Yang, 2018). There is still much work to be done to further optimize the innovation environment, and the direction of “innovation” needs to be firm. The innovation is always put at the core position of the overall development, and more innovation platforms are built to attract more innovative talents, apply more innovation results, thereby improving the economic innovation and competitiveness (Jiang et al., 2019).

As the core of technological innovation, enterprises are affected by various factors. Among them, employees are the key factors that determine the enterprise innovation efficiency. For ordinary employees in the enterprise, they are the creators who turn innovative thinking into reality. Without the pragmatic commitment of employees, innovation is just a piece of paper and loses its real value (Wang et al., 2019). The senior managers of the enterprise are mainly responsible for the formulation and decision-making of the innovation plan, thereby controlling the innovation direction of the enterprise. For senior managers and ordinary employees, they all work together for effective innovation of the enterprise. But both groups can stimulate their innovation efficiency through salary incentives (Hu and Sun, 2019). The salary of senior managers in Chinese listed enterprises is public. A reasonable salary system is a guarantee for managers to complete their performance. An imperfect salary system will cause dissatisfaction among managers, and will also aggravate conflicts between managers and shareholders. Ordinary employees of the enterprise also have personal development goals and have their expectations for salary. Although the enterprise has the ability to meet the salary expectations of individual employees, the ordinary employee base is large. Thus, it is also a significant expense in terms of salary. According to recent statistics, the salary gap between the highest-paid senior managers and ordinary employees is even more than a thousand times (Mokhov, 2019).

The scientific and technological innovation power of an enterprise is a necessary ability to maintain market competitiveness and maximize economic benefits. The senior managers of enterprises are the creators and decision-makers of innovative thinking. Ordinary employees are the finishers of innovative activities. Reasonable salary incentives for them play an important role in improving the innovation efficiency of enterprises. Entrepreneurs need to have a certain strategic direction when making incentive decisions. From the perspective of entrepreneur psychology, the individual entrepreneurs’ non-linear creative ideas based on information processing reflect the cognitive structure and values of them (Giancola, 2012). In fact, entrepreneurs have limited cognitive abilities, and they usually influence decision-making under the effect of psychological activities.

Therefore, based on the analysis of entrepreneur psychology, this study will focus on the relationship between the salary gap and innovation, as well as explore the relationship between employee salary gap and enterprise innovation efficiency. In addition, the market competitiveness of the products is included in the research. Then, the effect of the salary gap of employees on the enterprise innovation under different market competition intensities is analyzed, which provides a reference for promoting employees’ innovation activities and improving the market competitiveness of enterprises.

## Literature Review

In the context of knowledge economy, human capital has become an important resource for enterprises to create wealth. A dynamic enterprise salary system is part of good corporate governance, inspiring employees to maximize their enthusiasm and creativity in their work. Salary is an important means to motivate senior managers. Compared with salary level, the effect of salary structure incentive is more obvious. Existing investigations on the impact of the salary gap between senior managers and employees is rich, but there is some controversy. The academic community initially conducted an analysis through a direct regression between the salary gap of senior managers and enterprise innovation performance. One view is that the smaller salary gap between senior managers and employees helps employees realize their value and a stronger team awareness. Also, they are more actively involved in organizational development. Another view is that the increase in the salary gap between senior managers and employees will reduce employee productivity or increase employee turnover rates, which will hurt enterprise performance (Chen et al., 2018). From the analysis of the dual roles of the intermediary effect and moderating effect of research and development investment, empirical research finds that equity incentives positively promote the improvement of enterprise innovation performance. The research and development investment plays an intermediary role in senior manager equity incentives and innovation performance. Also, it plays a moderating role in the impact of core employee equity incentives and innovation performance.

In research on equity salary of senior managers and innovation performance, the ownership structure also affects innovation behaviors and decision-making of senior managers. Laux (2015) analyzes the optimal equity salary package for senior managers in an environment facing professional attention. The results show that, based on the value of the company’s potential growth opportunities and senior managers’ concerns about being fired, senior managers have two scenarios of excessive investment risk and excessive conservatism. The best salary plan to encourage the discovery of innovative ideas is stock option incentives, and restricting stocks against excessive conservatism. In a survey of employees’ thoughts on salary, Ridge et al. (2015) found that employees in enterprises not only pay much attention to their salary but also are accustomed to comparing their salary with the salary of the same or superior managers in the organization. Finally, it was found that the salary level within the enterprise can produce positive incentive results, thereby enhancing the level of employees’ work efforts, and improving the enterprise’s innovation process and performance output. When employees think that their efforts and inputs have not received the due rewards in the enterprise salary system, they are prone to dissatisfaction and unfairness. Furthermore, they are unwilling to work in teams and damage the organization’s innovation process and performance output (Lo and Wu, 2016).

Summing up the above research, it can be seen that whether the senior managers or ordinary employees in the enterprise may change their motivation and willingness to produce innovative behaviors. Furthermore, the impact of the salary structure’s incentive effect on innovation performance may also change. Based on this research, an analysis is carried out from the perspective of entrepreneurial psychology, focusing on the relationship between the salary gap and innovation (Zheng et al., 2015). Also, the relationship between employee salary gap and enterprise innovation efficiency is explored.

## Materials and Methods

### The Role of Entrepreneur Psychology in Decision-Making

When entrepreneurs conduct decision-making behaviors, the psychological standard structure may reflect the essential characteristics of entrepreneur decisions, including conditional standards such as psychological expectations and psychological motivation. Psychological standards can form a satisfactory interval for entrepreneur decision, and entrepreneurs make behavioral decisions based on the fusion of psychological standards within this interval. Judging whether the final plan determined by the entrepreneur is reasonable and consistent with the strategic development plan of the enterprise, is related to the level of the entrepreneur’s will and personal pursuit. Also, it is necessary to determine whether the final plan is feasible and whether it can when making decisions, including pursuit, desire, and ambition. The psychological activities of entrepreneurs will generate driving forces to guide the direction of rational decision-making (Liu et al., 2017). In the enterprise’s overall decision-making, the determination of the salary plan is also an important decision that the entrepreneur needs to complete. The decision-making ability is the necessary quality for the entrepreneur to search for a decision-making plan.

However, under the influence of external environmental stimuli, the decision-making psychological structure of entrepreneurs will shift, which will cause risk decision-making behaviors to change accordingly. The different decision-making psychological structures also determine the direction of risk decision-making behaviors. When making risk decisions, some entrepreneurs choose greater risks in pursuit of higher returns, while others are more cautious to make decisions with low returns (Liu et al., 2019). In different environments, the criteria for the same entrepreneur to choose and judge specific decisions also change due to changes in the environment and human factors. Entrepreneur decision is mainly for some special decisions. Thus, they need to have a special and stable psychological structure. In the process of researching the psychological decision-making of entrepreneurs, two major factions have emerged: the first is to focus on the motivation of entrepreneurs, that is, the motivation of the entrepreneur’s emotional load; the second is to focus more on the role of cognitive factors in decision-making behaviors (Zhang et al., 2018). Entrepreneurs’ decision-making power is greatly significant to the enterprise, and its motivation comes from the entrepreneur’s autonomy and strong willpower. Therefore, entrepreneurs usually have the urge to prove themselves better than others. Only success can be trusted and valued (Liu and Chen, 2018). In enterprises, as decision-makers, entrepreneurs are more likely to be overconfident, mainly because of information asymmetry. Among the more confident people, their temperament is usually more concerned, and they are more likely to become enterprise decision-makers.

**FIGURE 1:**
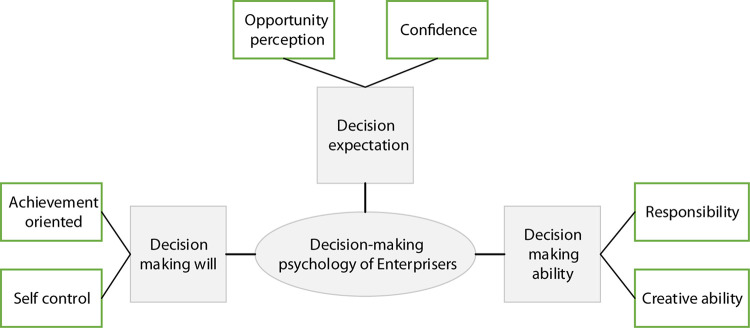
The specific structure of entrepreneur decision psychology.

Theories about the characteristics of enterprise leaders have been born in the 1930s. The psychological conditions and personal characteristics of enterprise leaders will be reflected in the life and practical activities (Goergen and Wood, 2014). In the eyes of the public, many entrepreneurs have shown good psychological qualities. However, deep in their hearts, there are many hardships and helplessness. Many enterprises have problems such as decision-making errors, interpersonal conflicts, and risk-taking, which have a lot to do with the psychological conditions of entrepreneurs. As a special group in modern Chinese society, the mental state of entrepreneurs typically reflects the psychological characteristics of the entire society. According to Freud, a perfect personality system consists of the id, the ego, and the superego. Self-achievement motivation has always been the focus of research on entrepreneur psychology. Based on individual achievement needs, the entrepreneur’s achievement motivation is regarded as a psychological process of individual pursuit of goals. Therefore, more emphasis is placed on the discussion of achievement motivation as a psychological characteristic (Abodohoui et al., 2013; Ye et al., 2018; Martínez et al., 2019). The reason for entrepreneurs to take risk action measures in the decision-making process is that they have relatively optimistic expectations about the set decision-making scheme. Also, the actual risk perceived by entrepreneurs is less than that perceived by most people who stand in an objective perspective, and capable entrepreneurs are more willing to take decision risks. To sum up, the psychological structure of entrepreneur decision is a three-factor two-dimensional structure, as shown in Figure 1.

### The Relationship Between Employee Salary Gap and Enterprise Innovation

Human capital is an important resource for enterprises to create value. In the past, senior managers were individuals who accepted commissions and acted as agents to manage the enterprise’s daily affairs. Due to the increasing complexity of the current enterprise structure, the needs of senior managers are more targeted. In addition to managing and arranging the day-to-day operations of the enterprise, they are also responsible for decision-making on major issues (Hah and Freeman, 2015). As the main body of the enterprise’s innovation decision-making, the senior managers are responsible for leading the entire process of enterprise innovation activities. If senior managers responsible for innovation activities lack enthusiasm, it will affect the implementation of innovation work, and the innovation ability of them will not be effectively exerted. Therefore, through appropriate incentives for employees, it can increase employees’ awareness of innovation investment and promote the technological innovation of enterprises, thereby maintaining the long-term stable development of enterprises in market competition (Fang and Shi, 2016; Kong et al., 2017). A systematic and dynamic salary system can ensure the orderly governance of the enterprise so that employees can gain their value and benefits while developing as much as possible, thereby motivating employees to maximize their enthusiasm in their daily work.

The study on the relationship between the monetary salary of senior managers and innovation performance found that senior managers are in the process of accumulating wealth in the short-term. At this time, the monetary salary will have a motivating effect on enterprise employees. Taking the data of innovative listed companies as a sample, the salary of senior managers helps to enhance their innovative motivation. As the total salary of higher-paid employees reaches a certain level, the stimulating effect of salary incentives on senior managers will be weakened (Feng and Peng, 2012). In addition, the change and redistribution of the shareholding structure will also have an effect on the innovative behavior of enterprise senior managers. Chinese scholars take the data of A-share listed companies from 2008 to 2016 as a research sample. The empirical analysis found that equity incentives can significantly promote the improvement of enterprise innovation performance. Also, adopting equity incentive policies for core employees of enterprises is conducive to the positive regulatory effect on innovation performance. From the perspective of shareholders, they pay more attention to the long-term development and value space of the enterprise. As a result, shareholders also tend to encourage enterprises to carry out innovative activities to a certain extent (Rajapathirana and Hui, 2018). However, for senior managers of enterprises, they pay more attention to whether they can obtain personal benefits during their tenure. Enterprise innovation activities are not only a high-risk project but also a measure to obtain higher returns. In the early stage, multiple investments of human and financial resources are usually required, and there will be lagging and uncertain returns. Therefore, some senior managers are not enthusiastic about enterprise innovation activities. On the contrary, the senior managers of enterprises are more interested in traditional controllable projects with short-term economic benefits.

The external incentive system can take into account the goals of both the enterprise and the employees to a certain extent. Finding such an incentive method can bring individual interests and the overall interests closer to each other and achieve incentive compatibility. It has been confirmed that the scientific application of incentive compatibility theory in enterprise governance has a positive effect on resolving conflicts of interest between enterprises and individual employees (Wu et al., 2019). By allowing individual employees to embody value in their work while obtaining corresponding returns, it can enhance the work creativity of employees, which means that the two objective functions of personal value and enterprise value are converged.

To sum up, academic circles have made it clear that the innovation consciousness of senior managers is an important factor to promote independent innovation of enterprises. Scientific salary incentives can affect the decision-making behavior of senior managers. Most of the current studies are based on the perspective of salary levels. It is generally believed that there is a positive correlation between the amount of salary and independent innovation. Entrepreneurs usually take innovation goals into consideration when formulating a salary system. Therefore, determining the salary structure of employees in enterprises is more worthy of exploration for promoting innovation.

### Research Hypotheses

Senior managers are the makers and guides of enterprise strategic goals, as well as promoters of innovation activities in enterprises. As a result, their innovation power plays an important role in driving the overall independent innovation of the enterprise. Monetary salary is the most direct incentive for senior managers, which can meet the material needs of them. The level of salary will change the work enthusiasm of senior managers accordingly. The working status of senior managers will affect the enthusiasm of ordinary employees at the grassroots level, so monetary salary incentives can connect the personal interests of senior managers with the interests of the company to stimulate them to actively innovate. Also, it can stimulate the work motivation of ordinary employees to give play to their personal value. According to Maslow’s demand theory, ordinary employees cannot participate in the decision-making and strategic planning of the senior management of the enterprise. Therefore, what affects their work status is the specific implementation of salary. If the salary of the grassroots employees in the enterprise is proportional to the labor pay of them, the employees’ enthusiasm can be fully mobilized. The long-term development of an enterprise requires not only high-level leaders with innovative scientific decision-making capabilities but also the executive coordination of ordinary employees. This reasonable allocation of human capital is an important prerequisite for enterprise development. Based on this, the hypotheses proposed in this paper are as follows.

H1: The salary of senior managers has a positive effect on innovation efficiency.
H2: The salary of ordinary employees has a positive effect on innovation efficiency.

At present, the senior management team becomes more complex with changes in the enterprise structure, and members of the senior management can be regarded as competitors. If the enterprise can clearly define the salary level in the senior management team and manage them from different job levels so that the salary of managers in different positions will increase with the increase of the level (Deng et al., 2019). In this way, the contribution of senior managers to the development of the enterprise can also produce a positive push. The stronger the working ability of senior managers is, the higher their promotion level will be. Correspondingly, there will be a gap with other senior managers in terms of salary, which can form a good competitive atmosphere in the team, thereby driving the members of the entire team to make innovation investments to promote innovation output (Wu et al., 2019). For ordinary employees, however, they are the executives of the innovative thinking of senior managers. If there is a large gap between the members who strive to achieve corporate innovation and the top management, it is easily will cause an unbalanced psychology of ordinary employees. If ordinary employees have dissatisfaction and no sense of belonging, it will kill the enthusiasm of the work and form a barrier between them and senior managers, hindering healthy communication within the enterprise (Li et al., 2019). Furthermore, the overall enthusiasm for innovation is suppressed, leading to a significant reduction in the innovation efficiency of enterprises (Meng, 2019). Therefore, the hypotheses proposed in this paper are as follows.

H3: Internal salary gap among senior managers have a positive effect on innovation performance.
H4: The salary gap between ordinary employees and senior managers has a negative effect on innovation performance.

### Study Sample Data and Variable Design

The salary system for most manufacturing enterprises in China is not perfect. At present, many enterprises improve economic benefits by reducing the labor costs of employees. Therefore, this paper selected the data of China’s A-share manufacturing listed companies from 2012 to 2016 as the research sample. This study excluded companies with abnormal or missing data in the sample, ST enterprises and companies with missing critical data to ensure the validity of the research results. Finally, a total of 1476 valid sample data were screened out, which were obtained through the CSMAR database and the Wande database. All continuous variables were subjected to 1% winsorize to avoid the effect of extreme values on the accuracy of the research results. Data collection and statistical analysis are performed on the original data samples using EXCEL 2016 and STATA14.0 software.

Explanatory Variables: (1) Senior management salary: This paper mainly studied the monetary salary and equity salary of senior managers. The annual salary was selected as the monetary salary. Equity salary can be expressed as: the number of shares held × year-end stock closing price. (2) Ordinary employee salary: The relative value method is used to measure, and it can be expressed as (total cash paid + payable salary at the end of the period-payable salary at the beginning of the period - senior management salary)/(number of employees-number of senior managers). (3) Salary gap: The salary gap within the senior management team is expressed as the average salary of the top three senior managers-the average salary of the senior management team. The salary gap between senior managers and ordinary employees is expressed as the natural logarithm of senior management salary/natural logarithm of ordinary employee salary.

Explained Variable: Enterprise innovation efficiency, specifically the number of patent applications by the enterprise.

Control Variables: (1) Enterprise size: generally, the larger the enterprise size, the higher the innovation efficiency of the enterprise. (2) Return on assets: the enterprise’s net profit/total assets. The greater the enterprise’s return on assets, the stronger the innovation ability. (3) Asset-liability ratio: total liabilities at the end of the year/total assets at the end of the year. The larger the enterprise’s asset-liability ratio, the lower the innovation efficiency. (4) Years of the establishment of an enterprise: generally, the level of enterprise management will increase with the extension of the establishment time of an enterprise. The longer the establishment time of an enterprise, the more resources it accumulates, and the stronger the innovation ability. (5) Dummy variables: four dummy variables are set in the 5-year period from 2012 to 2016. The dummy variable is an artificial variable used to reflect the qualitative attribute, and it is a quantified qualitative variable, usually taking the value 0 or 1.

The specific summary of explanatory variables, explained variables and control variables is shown in Table 1.

**TABLE 1:** Summary of variable design.

**Variable type**	**Variable name**	**Variable symbol**	**Variable definition and description**
Explanatory variable	Monetary salary of senior manager	Lngpay	Total Nian, logarithm
	Senior management salary	Lngequ	Number of shares held by senior manager × stock price at the end of the year, logarithm
	Ordinary employee salary	Lnppay	(total cash paid + payable salary at the end of the period - payable salary at the beginning of the period - senior management salary)/(number of employees-number of senior managers)
	Salary gap within the senior management team	Lnggap	Average salary of the top three senior managers - the average salary of the senior management team
	Salary gap between senior managers and ordinary employees	Lngygap	Average annual salary of senior managers/average salary of employees, logarithm
Explained variable	Enterprise innovation efficiency	Innovation	Number of patent applications
Control variable	Enterprise size	Size	Natural logarithm of total assets at the end of the period
	Return on assets	ROA	Net profit/Total assets of an enterprise to measure its profitability
	Asset-liability ratio	Leverage	Total liabilities at the end of the period/Total assets
	Years of establishment	Age	Natural logarithm of enterprise’s establishment time
	Dummy variable	Var	Set 4 dummy variables in the 5-year period from 2012 to 2016

### Model Building

Before constructing the econometric model in this study, the authors performed a correlation test between the variables to avoid multicollinearity. This paper used a multiple regression model to conduct an empirical analysis of the correlation between the salary gap and innovation efficiency. The regression function was used in data analysis in Excel. The range of independent and dependent variables was entered to obtain multiple regression results. β represents the coefficient of each variable, and ε represents the model residual.

(1) The relationship between the control variables and enterprise innovation efficiency was studied. The M1 model was constructed to test the effectiveness of the control model.

(1) $$I\,n\,n\,o\,v\,a\,t\,i\,o\,n_{i,t}=\beta_{0}+\beta_{1}\,S\,i\,z\,e_{i,t}+\beta_{2}\,R\,O\,A_{i,t}+\beta_{3}\,L\,e\,v\,e\,r\,a\,g\,e_{i,t}+\beta_{4}\,A\,g\,e_{i,t}+\beta_{5}\,V\,a\,r_{i,t}+\varepsilon$$

(2) The relationship between the salary gap and enterprise innovation efficiency was studied. After controlling related variables, M2–M6 were constructed to analyze the effect of the monetary salary and equity salary of senior managers, the ordinary employee salary, the internal salary gap of the senior management team, as well as the salary gap between senior managers and ordinary employees on the enterprise innovation efficiency.

(2) $$I\,n\,n\,o\,v\,a\,t\,i\,o\,n_{i,t}=\beta_{0}+\beta_{1}\,{\text{Lngpay}}_{i,t}+\beta_{2}\,S\,i\,z\,e_{i,t}+\beta_{3}\,R\,O\,A_{i,t}+\beta_{4}\,L\,e\,v\,e\,r\,a\,g\,e_{i,t}+\beta_{5}\,A\,g\,e_{i,t}+\beta_{6}\,V\,a\,r_{i,t}+\varepsilon$$

(3) $$I\,n\,n\,o\,v\,a\,t\,i\,o\,n_{i,t}=\beta_{0}+\beta_{1}\,{\text{Lngequ}}_{i,t}+\beta_{2}\,S\,i\,z\,e_{i,t}+\beta_{3}\,R\,O\,A_{i,t}+\beta_{4}\,L\,e\,v\,e\,r\,a\,g\,e_{i,t}+\beta_{5}\,A\,g\,e_{i,t}+\beta_{6}\,V\,a\,r_{i,t}+\varepsilon$$

(4) $$I\,n\,n\,o\,v\,a\,t\,i\,o\,n_{i,t}=\beta_{0}+\beta_{1}\,{\text{Lnppay}}_{i,t}+\beta_{2}\,S\,i\,z\,e_{i,t}+\beta_{3}\,R\,O\,A_{i,t}+\beta_{4}\,L\,e\,v\,e\,r\,a\,g\,e_{i,t}+\beta_{5}\,A\,g\,e_{i,t}+\beta_{6}\,V\,a\,r_{i,t}+\varepsilon$$

(5) $$I\,n\,n\,o\,v\,a\,t\,i\,o\,n_{i,t}=\beta_{0}+\beta_{1}\,{\text{Lnggap}}_{i,t}+\beta_{2}\,S\,i\,z\,e_{i,t}+\beta_{3}\,R\,O\,A_{i,t}+\beta_{4}\,L\,e\,v\,e\,r\,a\,g\,e_{i,t}+\beta_{5}\,A\,g\,e_{i,t}+\beta_{6}\,V\,a\,r_{i,t}+\varepsilon$$

(6) $$I\,n\,n\,o\,v\,a\,t\,i\,o\,n_{i,t}=\beta_{0}+\beta_{1}\,{\text{Lngygap}}_{i,t}+\beta_{2}\,S\,i\,z\,e_{i,t}+\beta_{3}\,R\,O\,A_{i,t}+\beta_{4}\,L\,e\,v\,e\,r\,a\,g\,e_{i,t}+\beta_{5}\,A\,g\,e_{i,t}+\beta_{6}\,V\,a\,r_{i,t}+\varepsilon$$

## Results

### Descriptive Statistics

**FIGURE 2:**
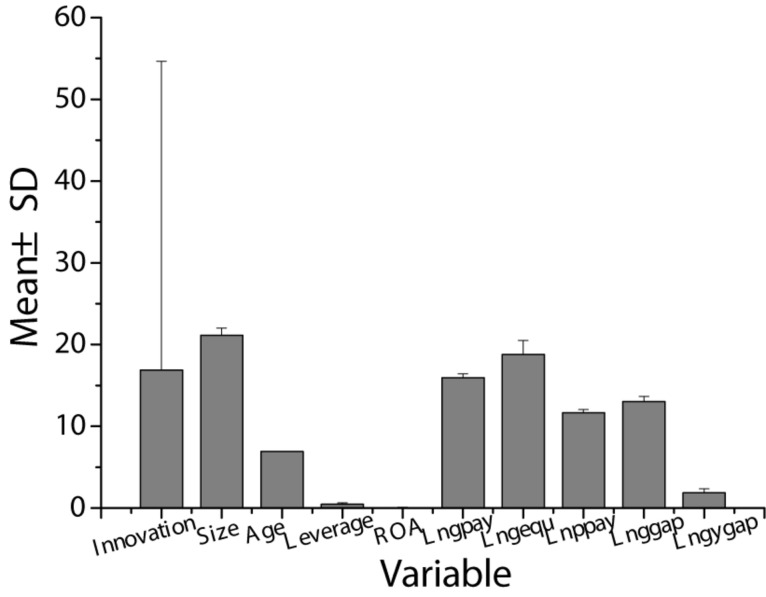
Descriptive statistical means of each variable.

This study performed a descriptive analysis of each variable, including maximum, minimum, mean value, and standard deviation. The specific results are shown in Table 2 and Figure 2. It can be seen that the mean value of enterprise innovation efficiency is 16.89, but the gap between the maximum and minimum is large. The minimum innovation efficiency of the enterprise is 0, indicating that there is a large difference in innovation efficiency between different enterprises in the manufacturing area. It may be related to the different areas targeted by each manufacturing enterprise. For example, some manufacturing industries that manufacture high-end technology products can maintain certain market competitiveness only by performing continuous technological innovation and obtaining more patents. For the control variables, the years of establishment of the enterprises are not much different, with a mean of 6.90. The mean size of the enterprise is 21.12, and the maximum is 24.52, and the minimum is 17.33. The mean asset-liability ratio of the enterprise is 0.44, as well as the maximum and minimum are 0.93 and 0.01, respectively. The difference between the two is large, indicating that there is a large difference in the solvency between manufacturing enterprises. The mean return on assets of the enterprise is 0.05, as well as the maximum and minimum are 0.18 and −0.88, respectively, which indicates that the return on assets of manufacturing enterprises varies greatly.

**TABLE 2:** Descriptive statistics of each variable.

**Variable**	***N***	**Mean value**	**Standard deviation**	**Maximum**	**Minimum**
Innovation	1476	16.89	37.76	178	0
Size	1476	21.12	0.89	24.52	17.33
Age	1476	6.90	0.04	7.32	5.76
Leverage	1476	0.44	0.20	0.93	0.01
ROA	1476	0.05	0.04	0.18	-0.88
Lngpay	1476	15.95	0.48	16.53	14.07
Lngequ	1476	18.77	1.75	23.25	14.21
Lnppay	1476	11.66	0.39	12.76	10.81
Lnggap	1476	13.03	0.62	14.39	11.76
Lngygap	1476	1.85	0.51	3.08	0.55

Among the explanatory variables, the mean monetary salary of senior managers is 15.95, and there is not much difference in the monetary salary of senior managers in the enterprise sample. The mean equity salary of senior managers is 18.77, and the standard deviation is 1.75. The extreme values are quite different, indicating that in terms of equity salary, there is an uneven situation among senior managers in various enterprises. The mean salary of ordinary employees is 11.66, and the difference between different enterprises is small. The mean salary gap between senior management teams is 13.03. The maximum and minimum are 14.39 and 11.76, respectively, indicating that there is a large difference in the internal salary gap of the senior management team of the enterprise, which is in an unbalanced state. The mean salary gap between senior managers and ordinary employees is 1.85, and the extreme values are quite different, which may be related to the differences in the internal resource allocation management of different enterprises.

### Correlation Analysis Results

The regression results of the correlation coefficients between salary, salary gap, and control variables are shown in Table 3. It can be seen that the monetary salary and equity salary of senior managers, the ordinary employee salary, and the internal salary gap of the senior management team are all significantly positively correlated at the level of 1% with the enterprise innovation efficiency. The correlation between the salary gap between senior managers and ordinary employees with enterprise innovation efficiency is not obvious. It confirms H1, H2, and H3 proposed earlier. The correlation coefficients of the regression between the variables are within the acceptable range, indicating that the model used does not have significant multicollinearity.

**TABLE 3:** Regression results of correlation coefficient of each variable (*n* = 1476).

**Variable**	**1**	**2**	**3**	**4**	**5**	**6**	**7**	**8**	**9**	**10**
Innovation	1									
Lngpay	0.209*	1								
Lngequ	0.104*	0.026	1							
Lnppay	0.141*	0.428*	0.043	1						
Lnggap	0.160*	0.818*	0	0.401*	1					
Lngygap	0.038	0.571*	–0.068	−0.270*	0.602*	1				
Leverage	−0.074*	0.077*	0.022*	–0.286	0.323*	0.551*	1			
ROA	0.097	0.167	–0.101	0.115	0.027*	0.011*	–0.073	1		
Size	0.239	0.471*	0.044	0.070	0.352*	0.309*	–0.300	0.459*	1	
Age	0.045	–0.049	0.031	–0.008	−0.077*	–0.050	–0.032	0.025	0.041	1

*Indicates a significant positive correlation at a level of 1%.

### Multiple Regression Results of Salary Gap and Innovation Efficiency

To further test the relationship between the variables, the effect of salary and salary gap on the enterprise innovation efficiency was studied. Model M1 is a regression test on the effectiveness of the control variables, and models M2–M6 are regression tests on the relevant variables of salary and salary gap. The multiple regression results between control variables and innovation efficiency have been listed in the previous text. The years of establishment of an enterprise has a positive correlation with innovation efficiency at the level of 1%. The longer the years of establishment of an enterprise, the more resources it accumulates. As a result, the investment of enterprises in innovation activities will be relatively large, promoting the improvement of innovation efficiency. The asset–liability ratio of the enterprise has a negative correlation with innovation efficiency. It is possible that a higher asset–liability ratio means higher debt and interest repayment. Therefore, decision-makers usually adopt a more insurable development strategy rather than higher-risk innovation activities, which is not conducive to the improvement of innovation efficiency.

**FIGURE 3:**
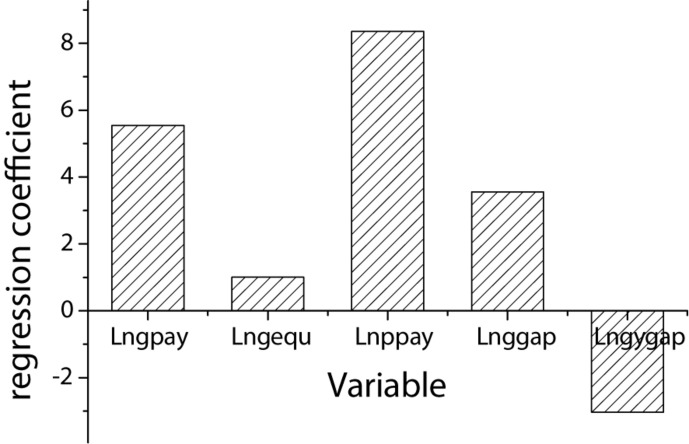
Multiple regression coefficient statistics of salary, salary gap and enterprise innovation efficiency.

The multiple regression results of salary, salary gap, and innovation efficiency are shown in Table 4 and Figure 3. It can be seen from Table 4 that the regression coefficients of monetary salary and equity salary of senior managers and enterprise innovation performance are 5.545 and 1.003, respectively, which are positively correlated at the level of 1%. It indicates that senior management salary has a positive effect on enterprise innovation efficiency, which validates H1. The regression coefficient of ordinary employee salary and enterprise innovation efficiency is 8.357, showing a positive correlation at the level of 1%. It indicates that ordinary employee salary has a positive effect on enterprise innovation efficiency, which validates H2. The regression coefficient between the internal salary gap of the senior management team and the enterprise innovation efficiency is 3.552, which is significantly positively correlated at the level of 1%. It indicates that the internal salary gap of the senior management team has a positive effect on enterprise innovation efficiency, which validates H3. The regression coefficient of the salary gap between senior managers and ordinary employees with the innovation performance of the enterprise is −3.032, which is significantly negatively correlated at the level of 5%. It indicates that the salary gap between senior managers and ordinary employees has a negative effect on the innovation performance of the enterprise, which validates H4.

**TABLE 4:** Multiple regression results of salary, salary gap, and enterprise innovation efficiency.

**Variable**	**Innovation**				
	**M2**	**M3**	**M4**	**M5**	**M6**
Lngpay	5.545*				
Lngequ		1.003*			
Lnppay			8.357*		
Lnggap				3.552*	
Lngygap					−3.032

## Discussion

If an enterprise wants to occupy a favorable position in today’s fiercely competitive market, it is an inevitable requirement to enhance its innovation ability. As the main body of innovation, manufacturing enterprises have played a key role in China’s economic development. How to promote enterprise innovation has become a topic of concern in various fields. But for enterprises, the implementation of innovation activities is usually more difficult, mainly due to the large risks of innovation activities and the uncertain economic benefits. As the creators and performers of innovative thinking, both senior managers and ordinary employees play an important role (Chin et al., 2019). Therefore, only by effectively motivating employees at all levels to work can it ensure the effective development of enterprise innovation activities. This paper explores the effect of employee salary and salary gap on enterprise innovation efficiency (Fadzil et al., 2019).

This paper selects the data of China’s A-share manufacturing listed enterprises from 2012 to 2016 as the research sample. The explanatory variables are the salary of senior management, the salary of the ordinary employee and salary gap. The explained variable is enterprise innovation efficiency. By constructing an econometric model, the authors use a multiple regression model to empirically analyze the correlation between the salary gap and innovation efficiency. The results show that the regression coefficients of monetary salary and equity salary of senior managers and enterprise innovation performance are 5.545 and 1.003, respectively. The regression coefficient between the salary of the ordinary employee and enterprise innovation efficiency is 8.357. The regression coefficient between the internal salary gap of the senior management team and the enterprise innovation efficiency is 3.552, both of which show a significant positive correlation at the 1% level. The regression coefficient between the salary gap between senior managers and ordinary employees and the enterprise innovation performance is −3.032, which is significantly negatively correlated at the 5% level. It can be concluded that the salary of senior managers, the salary of ordinary employees, and the internal salary gap of the senior management team have a significant positive effect on enterprise innovation efficiency. The salary gap between senior managers and ordinary employees has a negative effect on enterprise innovation efficiency.

From the perspective of incentive theory, the monetary salary of senior managers is used as an effective short-term incentive method, and equity salary is used as a long-term incentive method. It can meet the material needs and personal performance needs of senior managers, and stimulate their creative work, which is conducive to the promotion of enterprise innovation activities. Effective salary incentives for ordinary employees can enhance employees’ work enthusiasm and sense of enterprise belonging, thereby improving work efficiency in the implementation of innovative activities. The salary gap between senior managers can stimulate internal competition awareness, making them work together for enterprise innovation in a healthy competitive environment. However, the salary gap between ordinary employees and senior managers will amplify the psychological gap and imbalance of ordinary employees. It will affect their enthusiasm for work, further suppressing the innovation atmosphere of the enterprise.

## Conclusion

The internal salary gap of the senior management team has a significant positive effect on enterprise innovation efficiency, and the salary gap between senior managers and ordinary employees has a negative effect on enterprise innovation efficiency. In order to stimulate the work enthusiasm of employees at all levels and promote enterprise innovation, enterprises should optimize the salary structure from two levels – senior managers and ordinary employees. This research mainly focuses on the innovation efficiency of a wide range of manufacturing enterprises, yet some of them have a greater dependence on technological innovation in their operations. Therefore, in the subsequent research, the research subject can be refined into a special field of high-end technology product manufacturing enterprises, and the relationship between the salary gap and the enterprise innovation efficiency can be studied.

## Data Availability Statement

The raw data supporting the conclusions of this article will be made available by the authors, without undue reservation, to any qualified researcher.

## Ethics Statement

The studies involving human participants were reviewed and approved by Chongqing Institute of Engineering Ethics Committee. The patients/participants provided their written informed consent to participate in this study.

## Author Contributions

All authors listed have made a substantial, direct and intellectual contribution to the work, and approved it for publication.

## Conflict of Interest

The authors declare that the research was conducted in the absence of any commercial or financial relationships that could be construed as a potential conflict of interest.
